# Inhibition of BCL11B induces downregulation of PTK7 and results in growth retardation and apoptosis in T-cell acute lymphoblastic leukemia

**DOI:** 10.1186/s40364-021-00270-3

**Published:** 2021-03-04

**Authors:** Kehan Li, Cunte Chen, Rili Gao, Xibao Yu, Youxue Huang, Zheng Chen, Zhuandi Liu, Shaohua Chen, Gengxin Luo, Xin Huang, Grzegorz K. Przybylski, Yangqiu Li, Chengwu Zeng

**Affiliations:** 1 Key Laboratory for Regenerative Medicine of Ministry of Education, Institute of Hematology, Jinan University, Guangzhou, 510632 P.R. China; 2 Department of Hematology, First Affiliated Hospital, Jinan University, Guangzhou, 510632 P.R. China; 3 Department of Hematology, Guangdong General Hospital (Guangdong Academy of Medical Sciences), Guangzhou, 510080 P.R. China; 4 Institute of Human Genetics, Polish Academy of Sciences, Strzeszyńska 32, 60-479 Poznań, Poland

## Abstract

T-cell acute lymphoblastic leukemia (T-ALL) is an aggressive subtype of leukemia with poor prognosis, and biomarkers and novel therapeutic targets are urgently needed for this disease. Our previous studies have found that inhibition of the B-cell leukemia/lymphoma 11B (*BCL11B*) gene could significantly promote the apoptosis and growth retardation of T-ALL cells, but the molecular mechanism underlying this effect remains unclear. This study intends to investigate genes downstream of *BCL11B* and further explore its function in T-ALL cells. We found that *PTK7* was a potential downstream target of *BCL11B* in T-ALL. Compared with the healthy individuals (HIs), *PTK7* was overexpressed in T-ALL cells, and *BCL11B* expression was positively correlated with *PTK7* expression. Importantly, *BCL11B* knockdown reduced *PTK7* expression in T-ALL cells. Similar to the effects of *BCL11B* silencing, downregulation of *PTK7* inhibited cell proliferation and induced apoptosis in Molt-4 cells via up-regulating the expression of tumor necrosis factor (TNF)-related apoptosis-inducing ligand (*TRAIL*) and *p27*. Altogether, our studies suggest that *PTK7* is a potential downstream target of *BCL11B*, and downregulation of *PTK7* expression via inhibition of the *BCL11B* pathway induces growth retardation and apoptosis in T-ALL cells.

**Supplementary Information:**

The online version contains supplementary material available at 10.1186/s40364-021-00270-3.

**To the Editor:**

The B-cell leukemia/lymphoma 11B (*BCL11B*) gene plays an important role in the development of T-cell acute lymphoblastic leukemia (T-ALL) [1, 2]. Our previous studies have shown that down-regulation of *BCL11B* effectively inhibits proliferation and induces apoptosis of T-ALL cells [3, 4]. PTK7 (protein tyrosine kinase 7), the target protein of sgc8 DNA aptamer, has been identified as a potential biomarker for T-ALL [5]. However, the detailed role and downstream molecular mechanisms of *BCL11B* and relationship between *BCL11B* and *PTK7* remain undefined. In this study, we determined the *BCL11B* target genes in T-ALL patients using the Gene Expression Omnibus (GEO) database and used specific siRNAs (small interfering ribonucleic acid) to down-regulate the expression of this gene in T-ALL cell lines to explore the mechanism.

A total of 220 de novo T-ALL patients from the GEO database and 36 peripheral blood mononuclear cells (PBMCs) of T-ALL from our center were used for analysis and validation. In GSE13159 and GSE28497 datasets, we found that the *BCL11B* was highly expressed in T-ALL (*P* < 0.001, Fig. 1a and S1a). These results were consistent with our previous study [6]. Moreover, similar results of *PTK7* were found in GSE13159, GSE28497 and PBMCs (*P* < 0.05, Fig. 1a-b and S1b), which was also in line with previous report [7]. Next, to identify genes downstream of *BCL11B*, the *BCL11B* co-expression network was further characterized by weighted gene co-expression network analysis (WGCNA). Interestingly, Bioinformatics analysis [8] showed a significant positive correlation between the expression of *BCL11B* and *PTK7* in GSE13159, GSE28497 and PBMCs (*P* < 0.05, Fig. 1b-c and S1c). Furthermore, we studied the expression of *BCL11B* and *PTK7* in different cell lines from the Cancer Cell Line Encyclopedia (CCLE). Previous studies have shown that *BCL11B* is overexpressed in the acute type of adult T-cell leukemia/lymphoma (ATLL), and it is under-expressed in other lymphoma type. Consistently, *BCL11B* and *PTK7* was highly expressed in the T-ALL cells line but had low expression in the lymphoma cell lines (Fig. 1d-e). Based on these findings, we proposed that *PTK7* might be an important gene downstream of *BCL11B* in T-ALL.

**Fig. 1:**
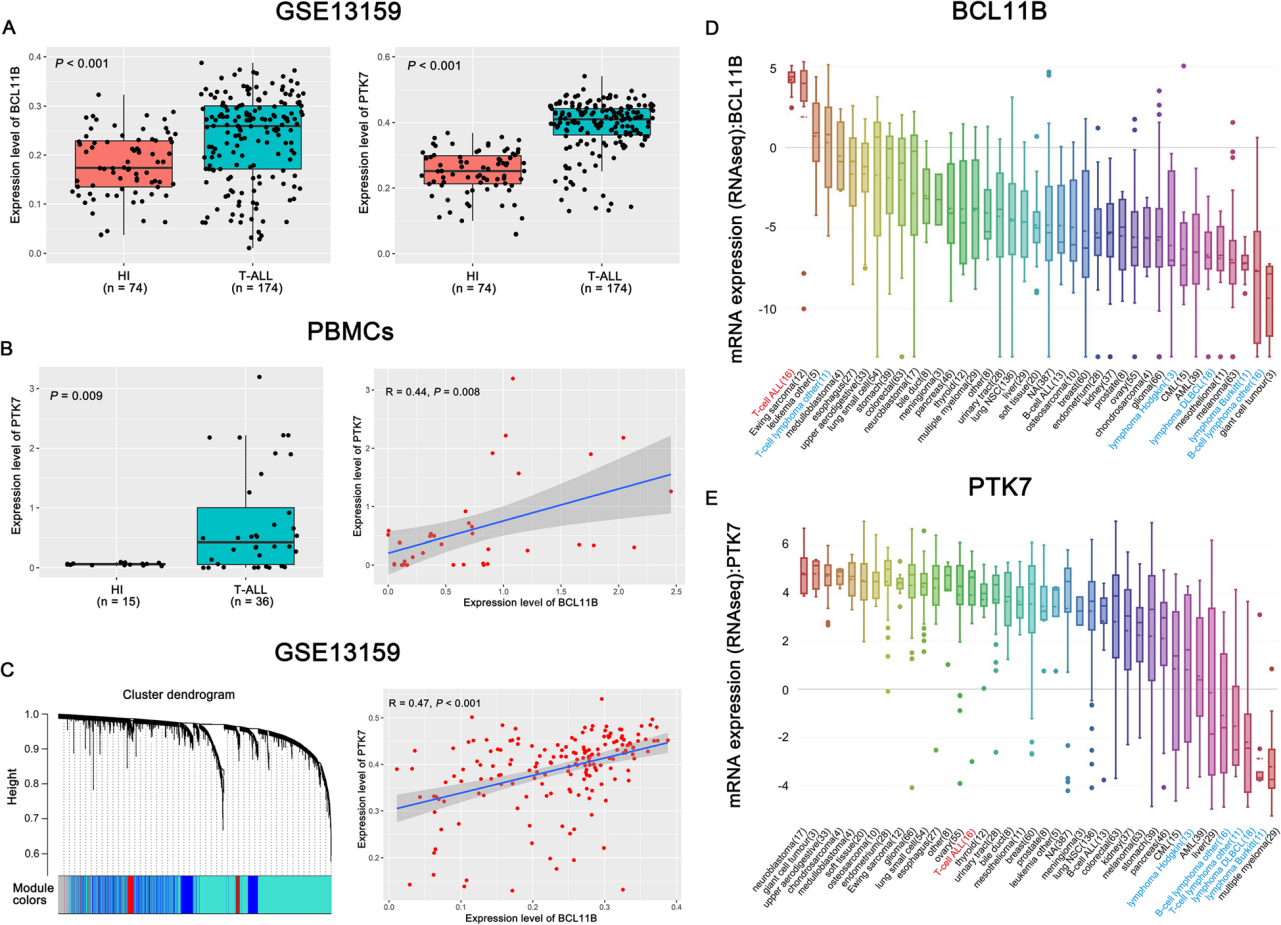
Positive correlation between *BCL11B* and *PTK7* in T-cell acute lymphoblastic leukemia (T-ALL). (**a**) The expression levels of *BCL11B* (left panel) and *PTK7* (right panel) in T-ALL patients and healthy individuals (HIs) in the GSE13159 dataset. (**b**) Validation of the correlation between *BCL11B* and *PTK7*. Expression levels of the *PTK7* gene in PBMCs from T-ALL patients and His (left panel). Linear correlation analysis of the *BCL11B* and *PTK7* genes in T-ALL samples (right panel). (**c**) *BCL11B* and *PTK7* are positively correlated in the GSE13159 dataset. Left panel: co-expression modules were obtained by weighted gene co-expression network analysis (WGCNA). The colored row beneath the dendrogram shows the module assignment determined by the Dynamic Tree Cut. A cluster dendrogram demonstrated that 10,459 differentially expressed genes were enriched in 11 co-expression network modules, while *BCL11B* and *PTK7* were located in the same blue module. Right panel: a positive correlation was detected between *BCL11B* and *PTK7*. Both *BCL11B* (**d**) and *PTK7* (**e**) are highly expressed in T-ALL compared to lymphoma cell lines. Data were obtained through the Cancer Cell Line Encyclopedia (CCLE). Detailed methods are available in Materials and Methods in Additional file 1

We sought to further verify the association between *BCL11B* and *PTK7* in both T-ALL and non-T-ALL cell lines at the mRNA and protein levels. As shown in Fig. 2a, *BCL11B* and *PTK7* mRNA were highly expressed in T-ALL cell lines (Jurkat and Molt-4), but almost absent in *BCL11B*-negative Burkitt lymphoma cell line (Raji). Next, an aptamer was used to determine the cell surface protein expression of *PTK7* in Jurkat and Raji cells. Sgc-8, the PTK7-specific aptamer, was labeled with a Cy5 fluorescent reporter and incubated with Jurkat and Raji cells under different concentrations, which revealed that Cy5-Sgc8 specifically bound to Jurkat cells but did not react with Raji cells (Fig. 2b). Excitingly, there was a significant decrease in *PTK7* mRNA expression after silencing *BCL11B* expression in the Jurkat and Molt-4 cells and cord blood (CB) CD3+ T cells (Fig. 2c-d). These results confirmed that *PTK7* might be regulated by the *BCL11B* signaling pathway in both T-ALL cell lines and human CD3+ T cells. Based on the above results, we attempted to further understand the role of *PTK7* in T-ALL cells. Compared to the negative control, the proliferation of Molt-4 cells transfected with *PTK7*-siRNA was significantly decreased (*P* < 0.01, Fig. 2e-f). Meanwhile, the Annexin V/PI-positive cells demonstrated a significant increase for *PTK7*-siRNA transfected Molt-4 cells, reaching 34.66 ± 5.21% (*P* = 0.008, Fig. 2g). In addition, recent reports have shown that tumor necrosis factor (TNF)-related apoptosis-inducing ligand (*TRAIL*) and *p27* are found to be involved in the *BCL11B* pathway to regulate the cell cycle and apoptosis [3, 9, 10]. Interestingly, significant increases in the expression levels of TRAIL and p27 were detected in the *PTK7*-siRNA group, which was consistent with the trend exhibited in the *BCL11B*-siRNA group (Fig. 2h). These data suggested that *PTK7* was a downstream *BCL11B* target gene in T-ALL cell growth and apoptosis.

**Fig. 2:**
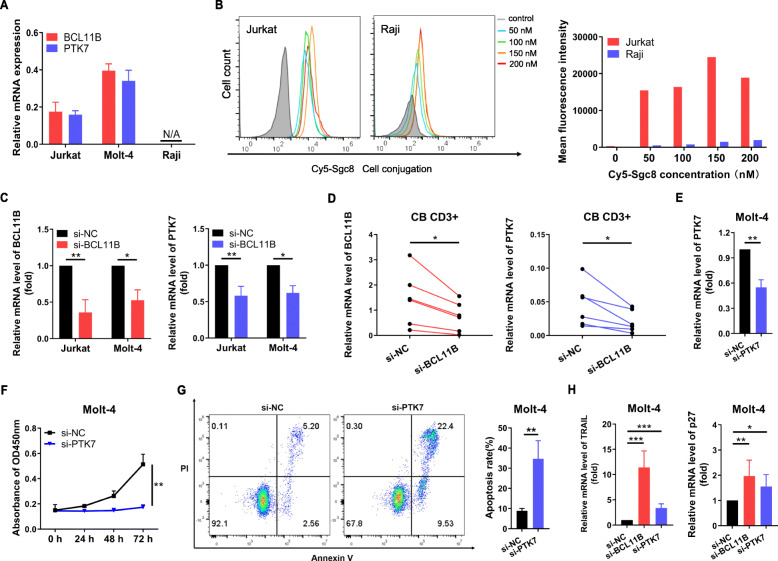
Downregulation of *PTK7* induces cell growth retardation and apoptosis. (**a**) The *BCL11B* and *PTK7* mRNA expression levels in T-ALL (Jurkat and Molt-4) and non-T-ALL (Raji) cell lines. *GAPDH* was used as an endogenous reference. (**b**) Cultured Jurkat and Raji cells were treated with Cy5-Sgc8 for 1 h at different concentrations. Flow cytometry analysis of the specific cell binding of Cy5-Sgc8 to Jurkat cells. (**c**) Expression of *PTK7* in T-ALL cell lines 48 h after *BCL11B* siRNA transfection. (**d**) Effect of *BCL11B* siRNA on the *BCL11B* mRNA expression level by real-time quantitative polymerase chain reaction (qRT-PCR) 48 h after transfection in CB CD3+ T cells. Negative control siRNA-treated cells were used for comparison. (**e**) Knockdown efficiency in Molt-4 cells were analyzed after knocking down the *PTK7* gene. (**f**) Molt-4 cells were transfected with si-*PTK7* and then cell proliferation was assessed by Cell counting kit-8 (CCK-8) assay. (**g**) Apoptosis in Molt-4 cells transfected with si-PTK7 was measured by flow cytometry after 48 h. (**h**) Expression of the *TRAIL* and *p27* genes in Molt-4 cells 48 h after transfected with *BCL11B* siRNA and *PTK7* siRNA. Non-specific siRNA treated cells were used as negative control. Asterisks signify statistically significant differences (***, *P* < 0.001; **, *P* < 0.01; *, *P* < 0.05)

In summary, this is the first report demonstrating that *PTK7* is an important functional downstream target gene of *BCL11B* in T-ALL. Our study provides rationale for targeting *BCL11B/PTK7* in the development of therapeutics for T-cell malignancies.

## Supplementary Information

**Additional file 1.** Materials and Methods.

**Additional file 2: Figure S1.** Expression patterns of *BCL11B* and *PTK7* in the GSE28497 dataset. High expression of *BCL11B* (A) and *PTK7* (B) in T-ALL. (C) *BCL11B* and *PTK7* had a positive correlation.

## Data Availability

Data available on request.

## Abbreviations

BCL11B: B-cell leukemia/lymphoma 11B
CCLE: Cancer cell line encyclopedia
GEO: Gene expression omnibus database
HI: Healthy individual
PTK7: Protein tyrosine kinase receptor 7
PBMCs: Peripheral blood mononuclear cells
siRNA: Small interfering ribonucleic acid
T-ALL: T-cell acute lymphoblastic leukemia
TRAIL: Tumor necrosis factor (TNF)-related apoptosis-inducing ligand
WGCNA: Weighted gene co-expression network analysis
