# Supplementary material for: Inhibition of BCL11B induces downregulation of PTK7 and results in growth retardation and apoptosis in T-cell acute lymphoblastic leukemia
Source: Biomark Res. 2021 Mar 4;9:17. doi: 10.1186/s40364-021-00270-3 (PMC7934371; doi:10.1186/s40364-021-00270-3)
Supplement: Supplementary file 1 — Additional file 1. Materials and Methods. [file 40364_2021_270_MOESM1_ESM.docx]

**Additional file 1**

**Materials and Methods**

**Gene Expression Omnibus (GEO) datasets**

A total of 174 and 46 RNA-seq data from patients with T-ALL in the GSE13159 [1] and GSE28497 [2], respectively, were downloaded from the GEO database (https://www.ncbi.nlm.nih.gov/geo/). Moreover, the corresponding RNA-seq data of 74 T-ALL patients and 4 healthy individuals (HIs) were acquired. The R limma package was used for differential gene expression analysis of 174 T-ALL patients and 74 HIs in GSE13159, and the screening criteria included a P < 0.001 and false discovery rate (FDR) < 0.001. After deleting duplicates, a total of 10 459 differential genes were used for weighted gene co-expression network analysis (WGCNA). The GEO datasets are publicly available; thus, approval from the local ethics committee was not required.

**WGCNA**

WGCNA can construct a scale-free network between genes, which is usually used to explore the expression patterns and functions of genes in cancer patients [3]. WGCNA was constructed using the R WGCNA, reshape2, and stringr packages. In the network, we use Pearson coefficient and soft-threshold to evaluate the weighted co-expression relationship between all genes in the adjacency matrix. Through hierarchical clustering in the adjacency matrix, a group of genes with high correlation are clustered into the same module, which is represented by different colors [4].

**Samples**

Peripheral blood (PB) samples from 36 de novo T-ALL patients were obtained from our center, including 25 males and 11 females, whose ages ranged from 3 to 80 years, and the median age was 28 years. Moreover, PB samples from 15 HIs were collected as a control. Peripheral blood mononuclear cells (PBMCs) were collected before treatment and separated using the Ficoll-Hypaque gradient centrifugation method. RNA was extracted using TRIzol reagent (Ambion, ThermoFisher Scientific, California, USA), and it was reverse transcribed into cDNA using Applied Biosystems™ High-Capacity cDNA Reverse Transcription Kit (ThermoFisher Scientific, California, USA) according to the manufacturer's instructions. All samples were obtained with informed consent, and this study was approved by the Ethics Committee of the Affiliated Hospitals of Jinan University.

**Cell culture**

Cells from the human T-ALL cell lines Jurkat and Molt-4 were obtained from American Type Culture Collection (ATCC, Manassas, Virginia, USA), and they were grown in RPMI 1640 medium (Gibco BRL, Grand Island, NY, USA) with 10% fetal calf serum (Gibco BRL, Grand Island, NY, USA). Cord blood (CB) CD3+ cells sorted by magnetic beads (Miltenyi Biotec) were maintained in GT-T551 H3 medium (Takara, Tokyo, JPN) with 10% fetal calf serum (Gibco BRL, Grand Island, NY, USA). All cells were maintained in a humidified incubator at 37°C and 5% CO_2_.

**RT-qPCR**

Real-time quantitative polymerase chain reaction (RT-qPCR) assays were used to detect the expression levels of BCL11B and PTK7 using the CFX96 Fast real-time PCR system (Bio-Rad, Hercules, California, USA). The amplification profile included initial denaturation at 95°C for 15 min followed by 45 cycles of denaturation at 95°C for 15 s, annealing at 64°C for 60 s, and extension at 64°C for 60 s [5]. The expression levels of the genes were normalized to GAPDH. The primer sequences are listed in Table 1.

**Flow cytometric analysis of cells bound with aptamer**

Aptamers are synthetic single-stranded DNA or RNA sequences that are selected to specifically recognize a molecular target with high affinity. Sgc8 is a DNA aptamer against the PTK7 receptor. Aptamer Sgc8 labeled Cy5 was synthesized by Shanghai Biological Engineering Technology Services Co., Ltd. and selected by HPLC purification. Half a million cells were incubated with Cy5-Sgc8 at the concentrations of 0 nM, 50 nM, 100 nM, 150 nM, and 200 nM in 200 μL binding buffer (0.2% BSA containing 5 mM MgCl_2_ and 20% FBS) for 1 h on ice. After washing the cells twice, the fluorescence for each cell sample was measured by flow cytometry (Cytoflex, Beckman Coulter, California, USA).

**RNA interference**

A validated specific siRNA directed against *BCL11B* and *PTK7* were obtained from RiboBio (Guangzhou, China). Non-targeting siRNA (siN0000001-1-5) was used as negative control. The siRNA oligos were transfected into Molt-4 cells by using the Neon® Transfection System (Invitrogen Neon MPK5000, ThermoFisher Scientific, California, USA). Transfections were performed as described previously [6-9].

**Cell proliferation and apoptosis assays**

Cell proliferation and apoptosis were determined using the cell counting kit-8 (CCK-8) assay (Dojindo, Tokyo, JPN) and flow cytometry (Cytoflex, BeckmanCoulter, California, USA), respectively, according to the manufacturer’s protocol. The percentage of apoptotic cells was confirmed by flow cytometry with Annexin V-FITC and PI staining (Multi Sciences, Hangzhou, CHN).

**Statistical analysis**

All statistical analysis was performed using R (version 3.6.1, https://www.r-project.org/) and SPSS software (version 13.0, Inc., Chicago, IL, USA). Differences in mRNA expression levels between T-ALL and healthy controls were tested by two independent-sample Wilcoxon tests. The paired-t test was used for comparison between the electric transfer group and control group. The Spearman method was used to analyze the correlation between the expression levels of BCL11B and PTK7. Differences with a two-tail P < 0.05 were considered statistically significant.

**References**

1. Kohlmann A, Kipps TJ, Rassenti LZ, Downing JR, Shurtleff SA, Mills KI, Gilkes AF, Hofmann WK, Basso G, Dell'orto MC, et al. An international standardization programme towards the application of gene expression profiling in routine leukaemia diagnostics: the Microarray Innovations in LEukemia study prephase. Br J Haematol. 2008; 142(5): 802-7.

2. Coustan-Smith E, Song G, Clark C, Key L, Liu P, Mehrpooya M, Stow P, Su X, Shurtleff S, Pui CH, et al. New markers for minimal residual disease detection in acute lymphoblastic leukemia. Blood. 2011; 117(23): 6267-76.

3. Chen CT, Wang PP, Mo WJ, Zhang YP, Zhou W, Deng TF, Zhou M, Chen XW, Wang SQ, Wang CX. Expression profile analysis of prognostic long non-coding RNA in adult acute myeloid leukemia by weighted gene co-expression network analysis (WGCNA). J Cancer. 2019; 10(19): 4707-18.

4. Zhang B, Horvath S. A general framework for weighted gene co-expression network analysis. Statistical applications in genetics and molecular biology. 2005; 4(Article17.

5. Chen C, Liang C, Wang S, Chio CL, Zhang Y, Zeng C, Chen S, Wang C, Li Y. Expression patterns of immune checkpoints in acute myeloid leukemia. J Hematol Oncol. 2020; 13(1): 28.

6. Wu Y, Hu Y, Yu X, Zhang Y, Huang X, Chen S, Li Y, Zeng C. TAL1 mediates imatinib-induced CML cell apoptosis via the PTEN/PI3K/AKT pathway. Biochemical and biophysical research communications. 2019; 519(2): 234-39.

7. Zeng C, Liu S, Lu S, Yu X, Lai J, Wu Y, Chen S, Wang L, Yu Z, Luo G, et al. The c-Myc-regulated lncRNA NEAT1 and paraspeckles modulate imatinib-induced apoptosis in CML cells. Mol Cancer. 2018; 17(1): 130.

8. Yu X, Hu Y, Wu Y, Fang C, Lai J, Chen S, Li Y, Zeng C, Zeng Y. The c-Myc-regulated miR-17-92 cluster mediates ATRA-induced APL cell differentiation. Asia Pac J Clin Oncol. 2019; 15(6): 364-70.

9. Zeng C, Xu Y, Xu L, Yu X, Cheng J, Yang L, Chen S, Li Y. Inhibition of long non-coding RNA NEAT1 impairs myeloid differentiation in acute promyelocytic leukemia cells. BMC Cancer. 2014; 14(693.

**Table 1.** Sequences of the primers, aptamer Sgc8 labeled Cy5, and siRNA.

| **Target** | **Sequences (5' - 3')** |
| --- | --- |
| *GAPDH* (forward) | AAGGTCGGAGTCAACGGATT |
| *GAPDH* (reverse) | CTGGAAGATGGTGATGGGATT |
| *BCL11B* (forward) | ATGTCCCGCCGCAAACAGG |
| *BCL11B* (reverse) | GGCTCGGACACTTTCCTGAGC |
| *PTK7* (forward) | GCTGGTGTTGGCCAATATTGC |
| *PTK7* (reverse) | AAGTAGCCGGGTTTGCCCT |
| Sgc8 aptamer labeled Cy5 | Cy5-ATCTAACTGCTGCGCCGCCGGGAAAATACTGTACGGTTAGA |
| si-*BCL11B* (sense) | GCACAACATGCAAGCAGCCCTTCAA |
| si-*PTK7* (sense)  si-NC | CGGGATGATGTCACTGGAGAA  cat. no. siN0000001-1-5 |

siRNAs were from RiboBio (Guangzhou, China).
