# Supplementary material for: Inhibition of BCL11B induces downregulation of PTK7 and results in growth retardation and apoptosis in T-cell acute lymphoblastic leukemia
Source: Biomark Res. 2021 Mar 4;9:17. doi: 10.1186/s40364-021-00270-3 (PMC7934371; doi:10.1186/s40364-021-00270-3)
Supplement: Supplementary file 2 — Additional file 2: Figure S1. Expression patterns of BCL11B and PTK7 in the GSE28497 dataset. High expression of BCL11B (A) and PTK7 (B) in T-ALL. (C) BCL11B and PTK7 had a positive correlation. [file 40364_2021_270_MOESM2_ESM.docx]

**Additional file 2**

**Figure S1**


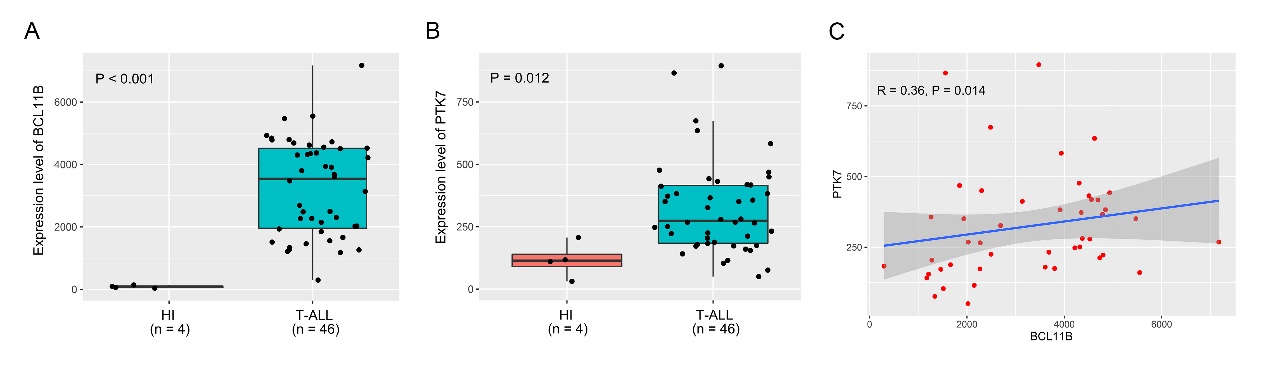


**Figure S1** Expression patterns of *BCL11B* and *PTK7* in the GSE28497 dataset. High expression of *BCL11B* (A) and *PTK7* (B) in T-ALL. (C) *BCL11B* and *PTK7* had a positive correlation.
